# Survival motor neuron protein and neurite degeneration are regulated by Gemin3 in spinal muscular atrophy motoneurons

**DOI:** 10.3389/fncel.2022.1054270

**Published:** 2022-12-22

**Authors:** Maria P. Miralles, Alba Sansa, Maria Beltran, Rosa M. Soler, Ana Garcera

**Affiliations:** Neuronal Signaling Unit, Experimental Medicine Department, Universitat de Lleida-IRBLleida, Lleida, Spain

**Keywords:** Gemin3, spinal muscular atrophy, survival motor neuron, motoneuron, NF-κB pathway, RelA

## Abstract

Spinal Muscular Atrophy (SMA) is a genetic neuromuscular disorder caused by reduction of the ubiquitously expressed protein Survival Motor Neuron (SMN). Low levels of SMN impact on spinal cord motoneurons (MNs) causing their degeneration and progressive muscle weakness and atrophy. To study the molecular mechanisms leading to cell loss in SMN-reduced MNs, we analyzed the NF-κB intracellular pathway in SMA models. NF-κB pathway activation is required for survival and regulates SMN levels in cultured MNs. Here we describe that NF-κB members, inhibitor of kappa B kinase beta (IKKβ), and RelA, were reduced in SMA mouse and human MNs. In addition, we observed that Gemin3 protein level was decreased in SMA MNs, but not in non-neuronal SMA cells. Gemin3 is a core member of the SMN complex responsible for small nuclear ribonucleoprotein biogenesis, and it regulates NF-κB activation through the mitogen-activated protein kinase TAK1. Our experiments showed that Gemin3 knockdown reduced SMN, IKKβ, and RelA protein levels, and caused significant neurite degeneration. Overexpression of SMN increased Gemin3 protein in SMA MNs, but did not prevent neurite degeneration in Gemin3 knockdown cells. These data indicated that Gemin3 reduction may contribute to cell degeneration in SMA MNs.

## Introduction

Spinal Muscular Atrophy (SMA) is an autosomal recessive neuromuscular disease characterized by the loss of function of spinal cord motoneurons (MNs) leading to muscle weakness and atrophy (Talbot and Tizzano, 2017). The disorder can be divided into five types, with the most severe showing onset at birth and the mildest having onset in adulthood. SMA patients have loss-of-function mutation or deletion in the *Survival Motor Neuron 1* (*SMN1*) gene and a nearly identical gene, *SMN2*, is always present (Wirth, 2021). *SMN1* and *SMN2* genes encode an identical protein, SMN, which is ubiquitously expressed and essential for cell survival. However, the predominant transcript from *SMN2* is truncated and lacks exon seven due to a single nucleotide change which affects its inclusion during the pre-mRNA splicing (Lorson et al., 1999; Monani et al., 1999). The truncated SMN protein is degraded and the limited full-length SMN protein expressed by *SMN2* is not sufficient to compensate for the *SMN1* deficit. The variable presentation of SMA in the clinic is largely explained by duplications and additions of SMN2 copies (Lefebvre et al., 1997). Inactivation of the single housekeeping *Smn* gene in mice causes early embryonic lethality (Schrank et al., 1997). Despite a broad spectrum of affected cellular processes, spinal cord MNs and neuromuscular junction are the primarily affected compartments in SMA (Sleigh et al., 2011).

Reduction in SMN protein affects many cellular and molecular pathways, including the biogenesis of the spliceosomal small nuclear ribonucleoproteins (snRNP) (Singh et al., 2017). SMN is part of a large macromolecular complex that functions in the assembly of spliceosomal small nuclear ribonucleoproteins (snRNP). The canonical SMN complex consists of SMN, Gemin 2-8, and Unrip (Pellizzoni et al., 2002; Blatnik et al., 2021). Additionally, SMN protein is present in the axonal compartment of MNs, where it is localized in granules that are actively transported into neuronal processes and growth cones. In these axonal granules, SMN is associated in protein complexes with Gemin2, Gemin3, and hnRNPR, but not with the spliceosomal Sm proteins, suggesting that its function in the axon may be distinct from those in the neuronal cell body (Zhang et al., 2006, 2008). These issues contribute to the hypothesis that SMN has specific functions affecting MNs related to the traffic of proteins and mRNAs down the MN axon modulating local protein translation during the formation and maintenance of neuromuscular junctions (Talbot and Davies, 2008; Blatnik et al., 2021).

The protein component of gems number 3 (Gemin3, a DEAD-box RNA helicase also known as DP103 or DDX20) is multifunctional, binding RNA secondary structures, regulating RNA metabolism, and performing other roles that do not directly involve RNA (Curmi and Cauchi, 2018). Gemin3 is a core member of the SMN complex together with Sm ribonucleoproteins and other Gemin proteins (Charroux et al., 1999). Additionally, Gemin3 can directly interact with several proteins including TAK1, a member of the MAPK family (Curmi and Cauchi, 2018). TAK1 is an upstream kinase of inhibitor of kappa B kinase beta (IKKβ); it functions as a cofactor enhancing TAK1-mediated IKKβ phosphorylation and consequently NF-κB activation (Shin et al., 2014). The canonical NF-κB pathway regulates cell survival and SMN protein expression in cultured mouse MNs (Mincheva et al., 2011), and it is compromised in SMA cultured mouse MNs (Arumugam et al., 2018).

In this study, we examined the NF-κB pathway and regulation of Gemin3 and SMN in several SMA models. Results clearly indicated that IKKβ, RelA, and Gemin3 were reduced in cultured SMA MNs and in SMA mouse postnatal spinal cord protein extracts. Nevertheless, when the level of these proteins was analyzed in non-neural SMA tissues, results showed differences from MNs observations. In SMA MNs, Gemin3, and SMN levels are interdependent: endogenous reduction of Gemin3 reduced SMN and vice versa. We also observed that endogenous reduction of Gemin3 induced neurite degeneration, which cannot be prevented by SMN over-expression. In summary, neurons and non-neuronal cells from SMA models displayed different molecular signs of intracellular deregulation and Gemin3 alterations observed in SMA MNs may contribute to understand some molecular mechanisms involved in SMA cell failure, such as neurite degeneration.

## Materials and methods

### SMA animals

Experiments involved two severe SMA mouse models, FVB⋅Cg-Tg (SMN2)^89Ahmb^Smn1^TM1Msd^/J (mutSMA) (RRID:IMSR_JAX:005024) and FVB⋅Cg-*Grm 7*^Tg(SMN2)89Ahmb^ Smn 1^TM1Msd^ Tg(SMN2*delta7)4299Ahmb/J (SMNdelta7) (RRID:IMSR_JAX:005025). mutSMA (Smn−/−; SMN2+/+) and SMNdelta7 (Smn−/−; SMN2+/+; SMNΔ7+/+) were obtained by crossing heterozygous (Smn±; SMN2+/+, and Smn±; SMN2+/+; SMNΔ7+/+, respectively) animals. Littermates mutSMA/SMNdelta7 and control (Smn+/+; SMN2+/+and Smn+/+; SMN2+/+; SMNΔ7+/+) were used for the experiments.

For MN purification or postnatal experiments, a fragment of the head or tail was obtained for genotyping. The REDExtract-N-Amp Tissue PCR Kit (Sigma, Darmstadt, Germany) was used for genomic DNA extraction and PCR setup, with the following primers: WT forward 5′-CTCCGGGATATTGGGATTG-3′, SMA reverse 5′-GGTAACGCCAGGGTTTTCC-3′, and WT reverse 5′-TTTCTTCTGGCTGTGCCTTT-3′. Birth was defined as postnatal day 0 (P0).

All procedures were done in accordance with the Spanish Council on Animal Care guidelines and approved by the University of Lleida Advisory Committee on Animal Services (CEEA02-01/17).

### Mouse MN isolation and culture

Motoneurons primary cultures were obtained from the spinal cords of CD1 or SMA mouse embryos at E13 essentially as described (Gou-Fabregas et al., 2009; Garcera et al., 2011). Isolated cells were pooled in culture medium and plated in laminin-coated four-well tissue culture dishes (Nunc, Thermo Fisher Scientific, Waltham, MA, USA) for either western blot analysis (60.000 cells/well) or neurite degeneration experiments (20.000 cells/well). Culture medium was Neurobasal medium (Gibco, Thermo Fisher Scientific, Waltham, MA, USA) supplemented with B27 (2% v/v; Gibco), horse serum (2% v/v; Gibco), L-glutamine (0.5 mM; Gibco), 2-mercaptoethanol (25 μM; Sigma), and a cocktail of neurotrophic factors, as follows: BDNF (1 ng/ml), GDNF (10 ng/ml), CNTF (10 ng/ml), and HGF (10 ng/ml) (Peprotech, London, UK). Twenty-four hours after plating, 2 μg/ml of aphidicolin (Sigma) was added to the culture medium and was maintained throughout the experiment.

Morphometric analysis of neurite degeneration was performed as described (Press and Milbrandt, 2008) with modifications. MNs were cultured and phase contrast microscopy images were obtained with a 20x lens at 12 days after plating. A grid was created over each image with NIH ImageJ software (Schneider et al., 2012), using the grid plugin (line area = 50,000). The cell-counting plugin was used to score each neurite. Degenerating and healthy neurites were counted in at least 10 fields per image of each well. Four different wells were counted for each condition and the experiments were repeated at least three times. Neurite segments were considered degenerated if they showed evidence of swelling and/or blebbing.

### Human fibroblasts cell lines culture

Human fibroblast cell lines were obtained from the Coriell Institute for Medical Research (Camden, NJ, USA). The Coriell Cell Repository maintains the consent and privacy of the donor samples. All cell lines and culture protocols in the present study were carried out under institutional review board guidelines at University of Lleida and the IRBLleida research center.

Two human fibroblast cell lines from patients with SMA (GM03813, SMAII; and GM09677, SMAI) and one unaffected control subject (GM03814, control) were purchased and cultured following manufacturer instructions. Cells were maintained in Eagle’s Minimum Essential Medium (EMEM) (Sigma) supplemented with non-inactivated fetal bovine serum (FBS; Gibco) (15% v/v), 0.5 M of L-Glutamine (Gibco), non-essential amino acids (Gibco) (1% v/v), and 20 μg/ml Penicillin-Streptomycin (Gibco). Cells were subcultured every 3–4 days. For western blot analysis, cells were plated at 3,000–4,000 cells/cm^2^ in 35 mm tissue-culture dishes and maintained in supplemented MEM. Two days later, total cell lysates were collected and submitted to western blot analysis.

### Differentiation of human-induced pluripotent stem cells (iPSCs) to MNs

The human iPSCs used in the present work were purchased from Coriell Institute for Medical Research or Cedars Sinai Biomanufacturing Center. The GM23411*B (Coriell) iPSC cell line (healthy non-fetal tissue) was considered the control condition, the GM23240*B (Coriell) iPSC cell line from a patient with SMA type 2 (2 copies SMN2; delta exon7-8 in SMN1) was considered the SMAII condition, and CS84iSMA-nxx (Cedars Sinai, Los Angeles, CA, USA) iPSC cell line from a patient with SMA type 1 (2 copies SMN2; delta exon7-8) was considered the SMAI condition. Control and SMA cells were differentiated to MNs as described (Du et al., 2015) with minor modifications (de la Fuente et al., 2020). Briefly, human iPSCs were cultured on a layer of irradiated mouse embryonic fibroblasts (MEFs) (Gibco) and neuroepithelial cells and motoneuron progenitors (MNPs) were generated. To induce MN differentiation, MNPs were detached with Accutase (Gibco) and cultured in suspension in MN induction medium (NEPIM plus 0.5 μM retinoic acid, 0.1 μM purmorphamine). Medium was changed on alternate days. After 6 days the neurospheres were dissociated and plated on laminin-coated plates in MN maturation medium (MN induction medium supplemented with 0.1 μM Compound E, Sigma, and 20 ng/ml ciliary neurotrophic factor, CNTF, and 20 ng/ml Insulin-like growth factor 1, IGF-1, both from Peprotech). Dissociated neurospheres were plated in laminin-coated four-well tissue culture dishes for western blot analysis (90,000 cells/well) or on 1 cm^2^ laminin-coated glass coverslips for immunofluorescence experiments (25,000 cells/well).

### Western blot analysis

Western blots were performed as previously described (Gou-Fabregas et al., 2009). For postnatal tissues analysis, pre-symptomatic P2 mutSMA and P5 SMAdelta7 mice were sacrificed by decapitation. Spinal cord lumbar regions 1 (L1) and 2 (L2), and gastrocnemius (distal muscle, analyzed in mutSMA) and quadriceps (proximal muscle, analyzed in SMAdelta7) (Kariya et al., 2008) were dissected and stored at –80^°^C. Samples were disaggregated using Direct Quant 100ST Buffer (DireCt Quant, Lleida, Spain) and a G50 Tissue Grinder (Coyote Bioscience, Alameda, CA, USA). Total cell lysates of cultured cells or tissue homogenates were resolved in SDS polyacrylamide gels and transferred onto polyvinylidene difluoride Immobilon-P transfer membrane filters (Millipore, Burlington, MA, USA), using an Amersham Biosciences (Slough, Buckinghamshire, UK) Semi-dry Trans-blot. The membranes were blotted with anti-SMN (1:5000; Cat. No. 610646, BD Biosciences, San Jose, CA, USA); anti-Gemin3 (12H12) (1:500; Cat. No. sc-57007) and anti-Gemin3 (D-5) (1:500; Cat. No. sc-271853) for human and mouse samples, respectively, both from Santa Cruz Biotechnology, Dallas, TX, USA; and anti-IKKβ (D30C6) (1:1000; Cat. No. 8943), and anti-NF-κB p65 (L8F6) (RelA) (1:1000, Cat. No. 6956), both from Cell Signaling Technology, Danvers, MA, USA. To control the specific protein content per lane, membranes were reprobed with monoclonal anti-α-tubulin antibody (1:50000; Cat. No. T5168, Sigma). Blots were developed using LuminataTM ForteWestern HRP Substrate (Millipore).

### Immunofluorescence

Cultured cells were fixed with 4% paraformaldehyde (Sigma) for 10 min and with cold methanol (Sigma) for 30 additional sec. Cells were permeabilized with 0.2% Triton X-100 and incubated for 2 h with 5% bovine serum albumin (BSA) in PBS. Primary antibody (anti-ChAT, 1:100, Cat. No. ab18736, Abcam; anti-Islet1/2, 1:50, Cat. No. 39.4D5, Developmental Studies Hybridoma Bank, Iowa City, IA, USA; anti-RelA, 1:200, Cat. No. 6956 and anti-βIIITubulin, 1:400, Cat. No. 5568, Cell Signaling Technology, Danvers, MA, USA; anti-Gemin3 12H12, 1:100; Cat. No. sc-271853, Santa Cruz Biotechnology; anti-HB9, 1:75, Cat. No. ab92606, Abcam, Cambridge, UK) was diluted in 0.2% Triton-X-100 and incubated overnight with 5% BSA in PBS. After washing, the secondary antibody was added: anti-mouse ALEXA555, 1:400, Cat. No. A21422 and anti-rabbit ALEXA488, 1:400, Cat. No. A11008, both from Invitrogen, Waltham, MA, USA and Cy™3 AffiniPure F(ab’)_2_ Fragment Donkey anti-Sheep IgG (H + L), 1:400, Cat. No. 713-166-147 from Jackson ImmunoResearch, West Grove, PA, USA. Hoechst (1:400, Sigma) staining was performed to identify nuclear localization in cell soma. Samples were mounted using Mowiol (Calbiochem, San Diego, CA, USA) medium. Microscopy observations were performed in a FV10i Olympus confocal microscope (Tokyo, Japan). Quantification of fluorescence intensity was performed blinded, using the NIH ImageJ software (Schindelin et al., 2012). Corrected Total Cell Fluorescence (CTCF) was measured selecting one cell at a time and measuring the area, integrated density, and mean gray value. The fluorescence intensity of each cell was calculated using the following formula: CTCF = Integrated Density (sum of the values of the pixels in the selection)—(area of selected cell × mean fluorescence of background readings). For each image, three background areas were used to normalize against autofluorescence.

### Plasmids and production of lentiviral particles

Lentiviral-based vectors for RNA interference-mediated gene silencing were performed as described (Garcera et al., 2011). For *SMN* interference, constructs were generated in pSUPER.retro.puro (OligoEngine, Seattle, WA, USA) using oligonucleotides that target the *SMN* sequence, indicated by capital letters as follows: shSMN, forward: gatccccCGACCTGTGAAGTAGCTAAttcaagagaTTAGCTACT TCACAGGTCGttttt, and reverse, agctaaaaaCGACCTG TGAAGTAGCTAAtctcttgaaTTAGCTACTTCACAGGTCGggg. Constructs were subcloned in pLVTHM vector containing the Green Fluorescence Protein (GFP). The shRNA lentiviral plasmids (pLKO.1-puro) for human/mouse Gemin3 were purchased from Sigma. The RefSeq used were NM_017397, corresponding to mouse Gemin3, and NM_007204, corresponding to human Gemin3. The clones TRCN0000009650 and TRCN0000007888 were designed for mouse and human interference of Gemin3, respectively.

Lentiviral particles were propagated in human embryonic kidney 293T (HEK293T) cells using the polyethylenimine (PEI, Sigma) cell transfection method. Plasmids containing the shRNA sequence or the empty vector, pSPAX2 and pM2G were cotransfected to HEK293T; lentivirus titer and MN transduction were performed as described previously (Garcera et al., 2011).

For SMN over-expression, the open reading frame of the human *SMN1* cDNA (NCBI accession number NM000344) was amplified by PCR from pDNR-LIB vector (Invitrogen) using the following primers, forward: cacaggatccatggcgatgagcagcggcggc; and reverse: tgtgggatccttaatttaaggaatgtgagca (underline indicates BamH1 restriction site) (Garcera et al., 2011). The PCR product was digested with *Bam*HI and cloned into FCIV plasmid (lentiviral expression vector containing Venus fluorescent protein, provided by Mario Encinas, Universitat de Lleida) to monitor transduction efficiency. Lentivirus containing over-expression constructs was obtained as described above.

### RNA isolation and quantitative RT-PCR

For qRT-PCR experiments, control and SMAII iPSCs were differentiated to MNs. Neurospheres were dissociated and plated in laminin-coated six-well tissue-culture dishes at a density of 400,000 cells/well in MN maturation medium. After 6 days, total RNA was extracted using the RNeasy^®^ Mini Kit (Qiagen, Hilden, Germany) according to manufacturer instructions. Eighty nanograms of total RNA from each condition were used for each individual qRT-PCR reaction. The assays were performed in a CFX96 Real-Time System (Bio-Rad, Hercules, CA, USA) using iTaq™ Universal SYBR^®^ Green One-Step Kit from Bio-Rad. Real-Time was performed using human RelA specific primers: Forward (5′-CGAGCTCAGTGAGCCCATG-3′) and Reverse (5′-GGCACAGCAATACGCCGGG-3′) or Gemin3 specific primers: Forward (5′-GCTGGCCGTTTTGGTACATTG-3′) and Reverse (5′-TGCACAGCAGCTTTAACTTCC-3′), and specific primers of human glyceraldehyde-3-phosphate dehydrogenase (GAPDH): forward (5′-TGCACCACCAACTGCTTAG-3′) and reverse (5′-AGAGGCAGGGATGATGTTG-3′) as internal control. Quantification was completed using Bio-Rad CFX Manager real-time detection system software (version 3.1, Bio-Rad). Each sample was measured in triplicate; relative fold change gene expression levels were calculated with the formula 2^^–(ΔΔCq)^.

### Statistical analysis

All experiments were performed at least three independent times. Values were expressed as mean ± estimated standard error of the mean (SEM). Statistical analysis was done with GraphPad Prism, version 8 (graphPad Software Inc.). Differences between groups were assessed by two-tailed Student *t*-test or one-way ANOVA with Tukey’s multiple comparisons or Dunnett’s multiple comparisons test for all other analysis. Similar variances between the compared groups were assumed. Values were considered significant when *p* < 0.05.

## Results

### IKKβ protein level reduction in SMA cells

**FIGURE 1:**
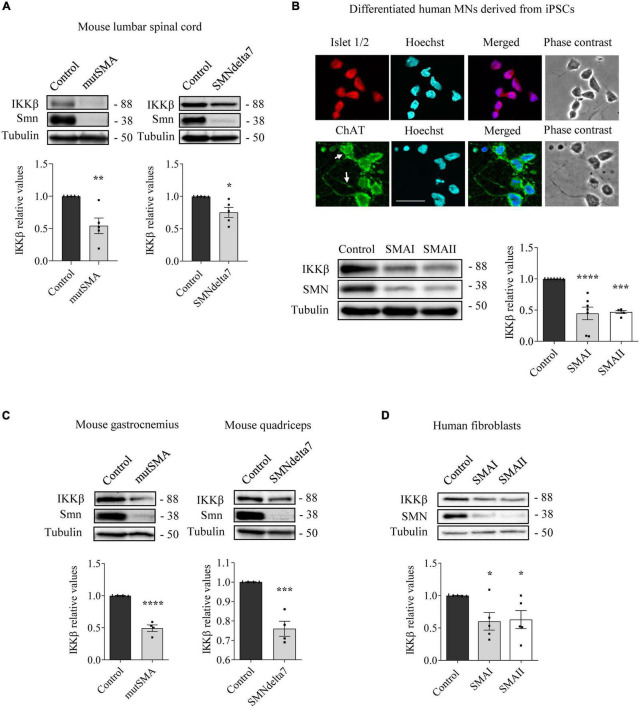
Reduced levels of inhibitor of kappa B kinase beta (IKKβ) protein in spinal muscular atrophy (SMA) models. **(A)** Total cell lysates of P2 control and mutSMA (left) or P5 control and SMNdelta7 (right) spinal cords were submitted to western blot analysis using an anti-IKKβ antibody. **(B)** Representative phase contrast and immunofluorescence images of 7 days differentiated human motoneurons (MNs) show the MN markers Islet1/2 (red) and ChAT (green). Hoechst (blue) staining was used to identify MN nuclei. Scale bar 40 μm. Protein extracts of 7 days differentiated control, SMAI and SMAII human MNs **(B)**, P2 control and mutSMA gastrocnemius and P5 control and SMNdelta7 **(C)**, and 48 h cultured control and SMAI and SMAII patient fibroblasts cell lines **(D)** were submitted to western blot using anti-IKKβ and anti-SMN antibodies. Membranes were reprobed using anti-α-tubulin antibody. Graph values represent the expression of IKKβ versus α-tubulin and correspond to the quantification of at least four independent experiments ± SEM. Asterisks indicate significant differences using Student *t*-test **(A,C)** or one-way Anova with Dunnett’s multiple comparisons post-test **(B,D)** (*p < 0.05, **p < 0.01, ***p < 0.001, ****p < 0.0001).

To analyze NF-κB pathway alterations in SMA condition, we assessed the levels of IKKβ protein in several SMA models. Overall, western blot analysis of SMA protein extracts showed reduced levels of IKKβ compared to non-SMA controls (Figure 1). Pre-symptomatic postnatal day 2 (P2) mutSMA and postnatal day 5 (P5) SMAdelta7 mice were genotyped; control (Smn+/+; SMN2+/+; and Smn+/+; SMN2+/+; SMNΔ7+/+, respectively) and mutant (mutSMA: Smn-/-; SMN2+/+; and SMNdelta7: Smn-/-; SMN2+/+; SMNΔ7+/+, respectively) animals were used for experiments. Spinal cords were dissected and protein extracts of lumbar regions 1 (L1) and 2 (L2) were submitted to western blot analysis. IKKβ protein level was significantly reduced in P2 mutSMA (0.54 ± 0.12, *p* = 0.0049) and in P5 SMNdelta7 (0.75 ± 0.078, *p* = 0.014) samples, compared to their respective controls (Figure 1A). SMA (SMAI from Cedars Sinai and SMAII from Coriell Institute) and non-affected control (from Coriell Institute) human iPSC cell lines were differentiated to MNs (Figure 1B) following the protocol as described (Du et al., 2015; de la Fuente et al., 2020). Immunofluorescence analysis showed that MN markers such as ChAT (Figure 1B) (95.42 ± 1.42% ChAT positive cells in SMA and control), Islet 1/2 (Figure 1B) (94.62 ± 1.37% Islet 1/2 positive cells in SMA and control), and HB9 (Figure 3B) (97.72 ± 0.86% HB9 positive cells in SMA and control) were expressed by these cells after 6 days in the presence of MN maturation medium, suggesting that human SMA and control iPSCs were properly differentiated to MNs. Protein extracts from 6 days differentiated SMA and control cultures were submitted to western blot. In both SMA cell cultures, IKKβ protein level was significantly reduced (SMAI 0.4471 ± 0.101, *p* < 0.0001, and SMAII 0.4693 ± 0.024, *p* = 0.0001) compared to the non-affected control (Figure 1B).

**FIGURE 2:**
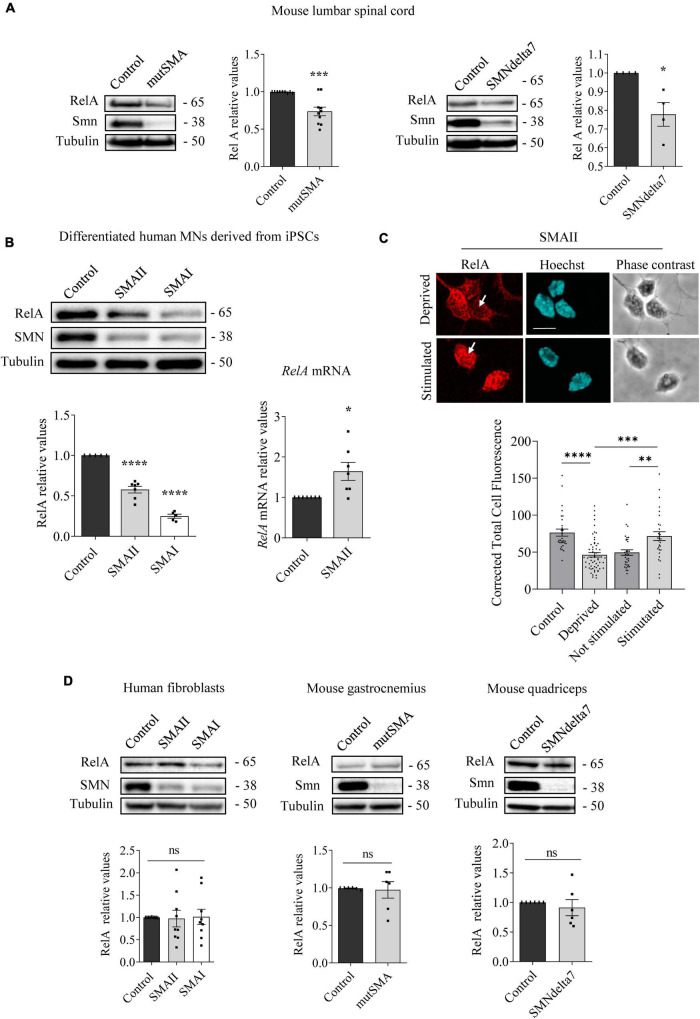
Changes of RelA protein level in spinal muscular atrophy (SMA) models. Western blot using anti-RelA and anti-SMN antibodies was used to analyze protein extracts of P2 control and mutSMA and P5 control and SMNdelta7 spinal cords **(A)**; 7 days differentiated control, SMAII, and SMAI human MNs **(B)** (left); 48 h cultured control, SMAII and SMAI human fibroblasts **(D)** (left); P2 control and mutSMA gastrocnemius and P5 control and SMNdelta7 quadriceps **(D)** (middle and right). Membranes were reprobed using anti-α-tubulin antibody. Graph values represent the expression of RelA versus α-tubulin and correspond to the quantification of at least four independent experiments ± SEM. **(B)** (right) Total RNA was extracted from 7 days differentiated control and SMAII human motoneurons (MNs) and reverse transcribed to cDNA. *Gapdh* gene was used as control. Graph values are the mean of *RelA* gene expression from three independent experiments ± SEM. **(C)** Seven days differentiated SMAII human MNs were cultured 24 h in the presence (control) or absence (deprived) of IGF and CNTF. Twenty-four hours later, cells were stimulated 5 min with medium containing (stimulated +) or not (stimulated –) 20 ng/ml IGF and 20 ng/ml CNTF. Cultures were fixed and processed for immunofluorescence using an anti-RelA antibody. Representative immunofluorescence confocal images of SMAII human MNs using an anti-RelA (red) antibody. Hoechst staining (blue) was used to identify MN nucleus. Graphs represent the mean of relative RelA fluorescence measured in MN nucleus, corresponding to the quantification of at least 30–40 cells per condition from three independent experiments ± SEM. Scale bar, 15 μm. Arrows indicate nuclear localization of RelA. Asterisks indicate significant differences using Student *t*-test **(A,B,D)** or one-way Anova with Dunnett’s or Tukey’s multiple comparisons post-test **(C,D)** (*p < 0.05; ^**^p < 0.005; ^***^p < 0.0005; ^****^p < 0.0001; ns, no significant differences *p* > 0.05).

**FIGURE 3:**
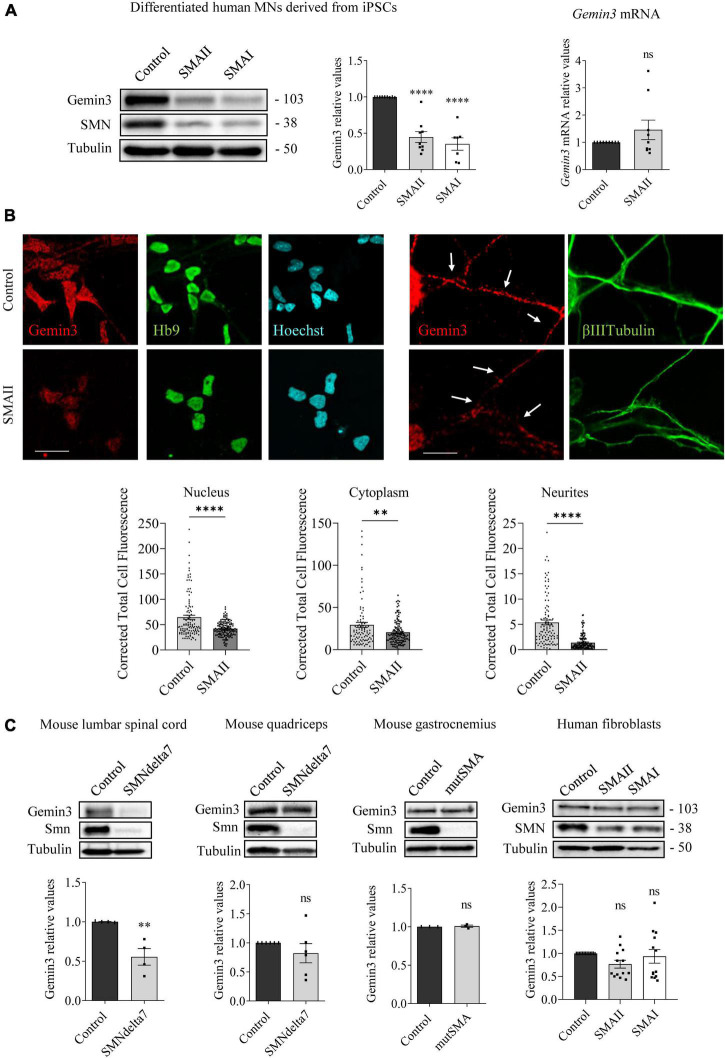
Gemin3 protein reduction in spinal muscular atrophy (SMA) human motoneurons (MNs) and mouse spinal cord. **(A)** Control and SMAII and SMAI MNs were differentiated for 7 days; (left) total cell lysates were obtained and submitted to western blot using anti-Gemin3 and anti-SMN antibodies. Membranes were reprobed using anti-α-tubulin antibody. Graph values represent the expression of Gemin3 versus α-tubulin and correspond to the quantification of at least seven independent experiments ± SEM; (right) total RNA was extracted and reverse transcribed to cDNA. *Gapdh* gene was used as control. Graph values are the mean of *Gemin3* gene expression from three independent experiments ± SEM. **(B)** Representative immunofluorescence images of 7 days cultures SMAII using anti-Gemin3 antibody (red), anti-HB9 antibody (green left), anti-βIIITubulin antibody (green right), and Hoechst staining (blue) to identify MN nucleus are shown. Graph values represent the mean of relative Gemin3 fluorescence measured in the cell nucleus, cytoplasm, and neurites, corresponding to the quantification of at least 100 cells per condition from three independent experiments ± SEM. Scale bar, 25 μm (left) and 15 μm (right). Arrows indicate MN neurites. **(C)** Total cell lysates of P5 control and SMNdelta7 spinal cords and quadriceps, P2 control and mutSMA gastrocnemius, and 48 h cultured control, SMAII, and SMAI patient fibroblasts, were submitted to western blot analysis using anti-Gemin3 and anti-SMN antibodies. Membranes were reprobed using anti-α-tubulin antibody. Graph values represent the expression of Gemin3 versus α-tubulin and correspond to the quantification of at least four independent experiments ± SEM. Asterisks indicate significant differences using Student *t*-test or one-way Anova with Dunnett’s multiple comparisons post-test (^**^p < 0.005; ^****^p < 0.0001 ns, no significant differences *p* > 0.05).

To analyze IKKβ alterations in SMA non-neural tissues, IKKβ protein level was assessed in mouse SMA muscle tissue and human SMA fibroblasts. Gastrocnemius (mutSMA) and quadriceps (SMNdelta7) were dissected from control and mutant genotyped mice at postnatal stage P2 (mutSMA) and P5 (SMNdelta7) and protein extracts were submitted to western blot analysis. IKKβ protein level was significantly reduced in P2 mutSMA (0.492 ± 0.051, *p* < 0.0001) and P5 SMNdelta7 (0.76 ± 0.038, *p* = 0.0008) extracts, compared to the corresponding controls (Figure 1C). Total protein cell lysates of 2 days cultured SMAI and SMAII fibroblasts (from Coriell Institute; see Section “Materials and methods”) were analyzed. The IKKβ protein level was significantly decreased in SMA fibroblasts (SMAI 0.602 ± 0.134, *p* = 0.0187; SMAII 0.628 ± 0.138, *p* = 0.028), compared to the clinical unaffected control (Figure 1D).

### RelA protein is reduced in human SMA differentiated MNs

To further explore modifications of NF-κB protein members in SMA, we next analyzed RelA levels. Total cell lysates of genotyped controls and P2 mutSMA or P5 SMNdelta7 lumbar spinal cords were submitted to western blot analysis using anti-RelA antibody. RelA protein level was significantly reduced in mutSMA (0.736 ± 0.058, *p* = 0.0006) and SMNdelta7 (0.778 ± 0.063, *p* = 0.013) protein extracts compared to their respective controls (Figure 2A). Protein extracts of 7 days differentiated human SMA and control MNs were obtained and submitted to western blot. RelA protein level was significantly reduced in SMAII (0.576 ± 0.04, *p* < 0.0001) and SMAI (0.248 ± 0.025, *p* < 0.0001) differentiated MNs compared to the non-affected control (Figure 2B). To determine whether the RelA reduction was associated with decreased activity of *RelA* gene expression we quantified *RelA* messenger RNA (mRNA) by quantitative RT-PCR (qRT-PCR). *Gapdh* gene was used as a control. SMAII and control iPSCs were differentiated to MNs. Total RNA from 7 days differentiated MNs was extracted and reverse-transcribed to cDNA, used as a template to quantify *RelA* transcript level. Results indicated that *RelA* mRNA expression (1.64 ± 0.22, *p* = 0.013) was increased in SMAII cells compared to control (Figure 2B). These observations suggested that RelA protein decrease in SMA differentiated MNs was not associated to reduced *RelA* gene expression.

After IKK and IκB phosphorylation during NF-κB pathway activation by cytokines, NF-κB complex is released, relocates to the nucleus, and binds to the κB DNA elements, resulting in gene induction or gene repression (Perkins, 2007). In this context, we wanted to evaluate whether cytoplasmic RelA was able to translocate to the nucleus after neurotrophic factor stimulation in SMA MNs. To this end SMAII MNs were differentiated for 7 days and then cultured 24 h in the presence (control) or the absence (deprived) of IGF (20 ng/ml) and CNTF (20 ng/ml) in the culture medium. Deprived cells were stimulated for 5 min with medium containing (stimulated +) or not (stimulated –) IGF (20 ng/ml) and CNTF (20 ng/ml). Using an anti-RelA antibody, immunofluorescence confocal images showed that 24 h deprived cells exhibited reduced Corrected Total Cell Fluorescence (CTCF) in SMA MN nuclei (46.37 ± 2.99, *p* < 0.0001), compared to the non-deprived control nuclei (76.39 ± 4.68). When 24 h deprived cells were stimulated in the presence or the absence of neurotrophic factors, fluorescence measures indicated that IGF and CNTF treatment induced nuclear relocation of RelA (CTCF: Stimulated – 49.45 ± 3.69; Stimulated + 71.66 ± 5.9, *p* = 0.0019) (Figure 2C). Together, these results suggest that RelA protein was reduced in SMA cells; nevertheless, when NF-κB pathway was stimulated by neurotrophic factors, RelA preserved its capability to translocate from the cytoplasm to the nucleus, suggesting activation of the pathway.

We next analyzed RelA in SMA cultured human fibroblasts, P2 mouse mutSMA gastrocnemius, and P5 mouse SMNdelta7 quadriceps. As shown in Figure 2D, no significant differences of RelA protein level were observed in SMA mouse muscle tissue (gastrocnemius 0.973 ± 0.11, *p* = 0.8101; quadriceps 0.911 ± 0.14, *p* = 0.5321) or SMA human fibroblasts (SMAII 0.973 ± 0.19, *p* = 0.8833; SMAI 1.012 ± 0.17, *p* = 0.9469), compared to their respective controls.

### Gemin3 protein is reduced in differentiated human SMA MNs

To study whether Gemin3 deregulation in SMA models is present in neurons and non-neuronal cells, we analyzed Gemin3 protein level by western blot and immunofluorescence using an anti-Gemin3 antibody. SMA and control human iPSC cells were differentiated to MNs and 7 days protein extracts were obtained. Gemin3 protein level was significantly reduced in human SMAII (0.447 ± 0.074, *p* < 0.0001) and SMAI (0.351 ± 0.085, *p* < 0.0001) MNs, compared to the control condition (Figure 3A). To explore whether the Gemin3 protein reduction in SMA condition was associated with decreased level of *Gemin3* transcript, we quantified by qRT-PCR *Gemin3* mRNA in 7 days differentiated control and SMAII MNs. *Gapdh* gene was used as a control. *Gemin3* mRNA was not reduced in SMAII cells (1.45 ± 0.36, *p* = 0.221) compared to control (Figure 3A). Since MNs are highly polarized cells, we selected immunofluorescence to observe Gemin3 protein in MN soma and neurites. After differentiation, SMAII and control cultures were fixed and processed using an anti-Gemin3 antibody. The CTCF was quantified using NIH ImageJ software. We observed a reduction of Gemin3 in nucleus, cytoplasm, and neurites of human SMAII differentiated MNs (CTCF: nucleus, 41.92 ± 1.29, *p* < 0.0001; cytoplasm, 20.5 ± 1.23, *p* = 0.0026; neurites, 1.39 ± 0.13, *p* < 0.0001), compared to control condition (CTCF: nucleus, 64.59 ± 4.15; cytoplasm, 29.35 ± 2.92; neurites, 5.38 ± 0.47) (Figure 3B). Together these results indicated a reduction of Gemin3 protein, but not *Gemin3* mRNA, in human differentiated SMA MNs. Next, we examined Gemin3 levels in protein extracts of P5 SMNdelta7 spinal cords and quadriceps, P2 mutSMA gastrocnemius, and 2 days cultured human SMA fibroblasts (Figure 3C). Results indicated that Gemin3 was significantly reduced in SMNdelta7 spinal cord (0.555 ± 0.105, *p* = 0.0055), compared to control condition (Figure 3C). Nevertheless, Gemin3 protein was not reduced in human SMA fibroblasts (SMAII 0.765 ± 0.085, *p* = 0.177; SMAI 0.934 ± 0.14, *p* = 0.856), in mouse SMNdelta7 quadriceps (0.821 ± 0.164, *p* = 0.302), or in gastrocnemius mutSMA (1.007 ± 0.014, *p* = 0.691), compared to their respective controls. These results together revealed divergences in Gemin3 protein expression in SMA cells and tissues.

### Gemin3 endogenous reduction regulates SMN, IKKβ, and RelA protein levels in cultured MNs

**FIGURE 4:**
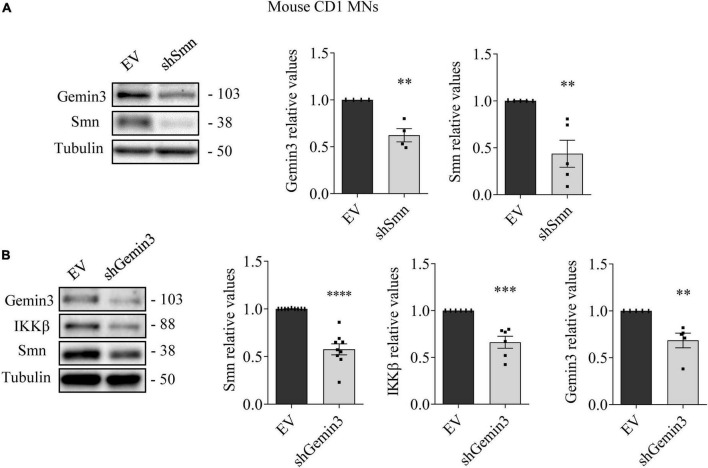
Survival motor neuron (SMN) and Gemin3 protein level inter-regulation in mouse motoneurons (MNs). Cultured mouse CD1 MNs were transduced with shSmn **(A)**, shGemin3 **(B)**, or empty vector (EV) **(A,B)** lentiviral constructs. Seven days after transfection, protein extracts were obtained and submitted to western blot using anti-Gemin3, anti-IKKβ, and anti-SMN antibodies. Membranes were reprobed using anti-α-tubulin antibody. Graph values represent the expression of Gemin3 **(A,B)**, IKKβ **(B)** or SMN **(A,B)** versus α-tubulin and correspond to the quantification of at least four independent experiments ± SEM. Asterisks indicate significant differences using Student *t*-test (^**^p < 0.01, ^***^p < 0.001; ^****^p < 0.0001).

To further explore SMN, NF-κB members, and Gemin3 protein level regulation in cultured MNs, we used shRNA to knockdown SMN or Gemin3 in mouse and human MNs. We generated shRNA sequences targeting specific sites of mouse Smn (Garcera et al., 2011) or of human or mouse Gemin3, and produced lentiviral particles containing the shRNAs (shSmn and shGemin3, respectively). We also generated a lentivirus containing an expression construct of the human SMN gene (ovSMN) (Garcera et al., 2011). MNs from 13.5 days CD1 embryos were isolated and transduced using the shSmn or shGemin3 or the control empty vector (EV) lentiviral particles. Seven days after transduction, protein extracts of CD1 MNs were collected and submitted to western blot using Gemin3, IKKβ or SMN antibodies. Immunoblot analysis of Smn and Gemin3 level displayed effective knockdown in shSmn (Figure 4A) and shGemin3 (Figure 4B) transduced CD1 MNs (Smn 0.407 ± 0.12, *p* < 0.0001; Gemin3 0.684 ± 0.078, *p* = 0.0038, respectively). Results indicated that shSmn treatment significantly reduced Gemin3 level (0.62 ± 0.07, *p* = 0.0033) (Figure 4A). Furthermore, shGemin3 addition significantly reduced Smn (0.576 ± 0.057, *p* < 0.0001) and IKKβ (0.661 ± 0.064, *p* = 0.0004) protein level, compared to their respective EV controls (Figure 4B).

**FIGURE 5:**
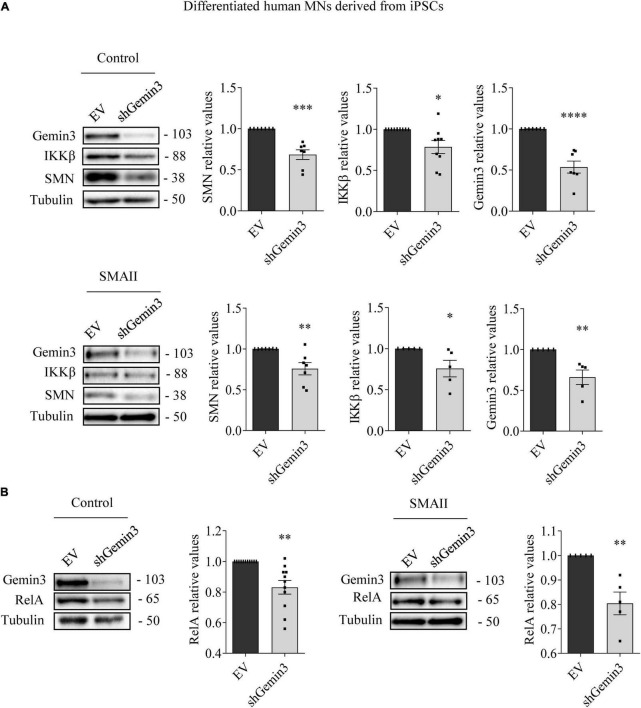
Gemin3 knockdown reduced survival motor neuron (SMN), IKKβ, and RelA protein level in differentiated human motoneurons (MNs). Control and SMAII human MNs were plated and transduced with lentivirus containing shGemin3 construct or the empty vector (EV). Protein extracts from 7 days transduced cells were submitted to western blot using anti-Gemin3, anti-IKKβ, anti-RelA, and anti-SMN antibodies. Membranes were reprobed using anti-α-tubulin antibody. Graph values represent the expression of Gemin3 **(A)**, SMN **(A)**, IKKβ **(A)**, or RelA **(B)** versus α-tubulin and correspond to the quantification of at least five independent experiments ± SEM. Asterisks indicate significant differences using Student *t*-test (*p < 0.05; ^**^p < 0.005; ^***^p < 0.0005; ^****^p < 0.0001).

**FIGURE 6:**
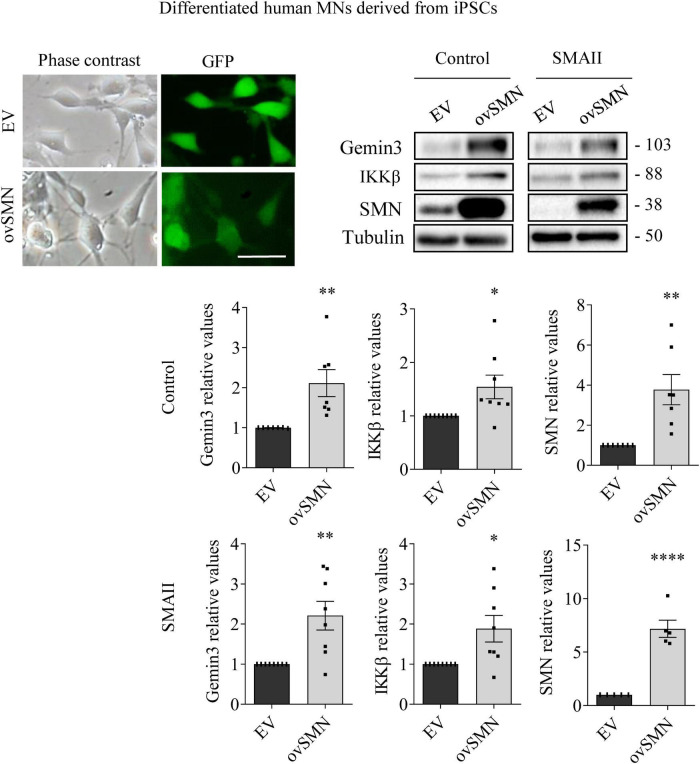
Survival motor neuron (SMN) overexpression increased Gemin3 and IKKβ protein level in differentiated human motoneurons (MNs). Control and SMAII human MNs were plated and transduced with lentiviral vectors carrying ovSMN construct or the empty vector (EV). Representative microscopy images of 7 days EV or ovSMN transduced cultures: phase contrast and GFP. GFP indicates Green Fluorescent Protein expressing cells in the same microscopic field. Scale bar, 20 μm. Total cell lysates were submitted to western blot analysis using anti-Gemin3, anti-IKKβ, and anti-SMN antibodies. Membranes were reprobed using anti-α-tubulin antibody. Graph values represent the expression of Gemin3, IKKβ or SMN versus α-tubulin and correspond to the quantification of at least five independent experiments ± SEM. Asterisks indicate significant differences using Student *t*-test (*p < 0.05; ^**^p < 0.01; ^****^p < 0.0001).

MN progenitors (MNPs) cells derived from human non-affected control and SMAII iPSCs were plated in MN differentiation medium and transduced with lentiviral particles containing shGemin3 (Figure 5) or ovSMN (Figure 6) or EV constructs. Twenty-four hours after transduction, culture medium was changed and fresh differentiation medium was added. Seven days later, total cell lysates were obtained and western blot analysis was performed using SMN, Gemin3, IKKβ, or RelA antibodies. As expected, Gemin3 protein was reduced in control and SMAII cells treated with shGemin3 (control: 0.535 ± 0.073, *p* < 0.0001; SMAII: 0.66 ± 0.089, *p* = 0.0051), compared to the EV transduced controls (Figure 5A). SMN protein level assessment on Gemin3-reduced cultures revealed a significant decrease of SMN in control (0.684 ± 0.058, *p* = 0.0002) and SMAII (0.757 ± 0.075, *p* = 0.0073) conditions (Figure 5A). When NF-κB members were analyzed in shGemin3 MNs, we observed a significant reduction of IKKβ (Figure 5A) and RelA (Figure 5B) protein level in control (IKKβ 0.784 ± 0.079, *p* = 0.014; RelA 0.83 ± 0.044, *p* = 0.0011) and SMAII (IKKβ 0.76 ± 0.1, *p* = 0.043; RelA 0.80 ± 0.046, *p* = 0.0028) cells, compared to their respective EV controls. This suggests a direct association between Gemin3 and SMN, IKKβ, and RelA level in cultured human MNs.

To explore whether SMN increase was able to counteract Gemin3 and IKKβ reduction in SMA MNs, control and SMAII iPSCs were differentiated to MNs and transduced with lentivirus containing ovSMN construct to increase SMN protein level in these cells. Protein lysates of 7 days transduced MNs were obtained and submitted to western blot using anti-Gemin3 or anti-IKKβ antibodies. SMN protein was significantly increased in ovSMN transduced control (3.773 ± 0.751, *p* = 0.0031) and SMAII (7.168 ± 0.804, *p* < 0.0001) cells compared to the EV condition, indicating that overexpression was accomplished. When Gemin3 and IKKβ were analyzed in ovSMN treated cultures, the protein level was significantly increased in control (Gemin3 2.111 ± 0.337, *p* < 0.0059; IKKβ 2.111 ± 0.337, *p* < 0.0059) and SMAII (Gemin3 2.209 ± 0.358, *p* = 0.0044; IKKβ 2.111 ± 0.337, *p* < 0.0059) cells, compared to their EV respective controls (Figure 6).

### SMN overexpression does not prevent neurite degeneration induced by Gemin3 knockdown

**FIGURE 7:**
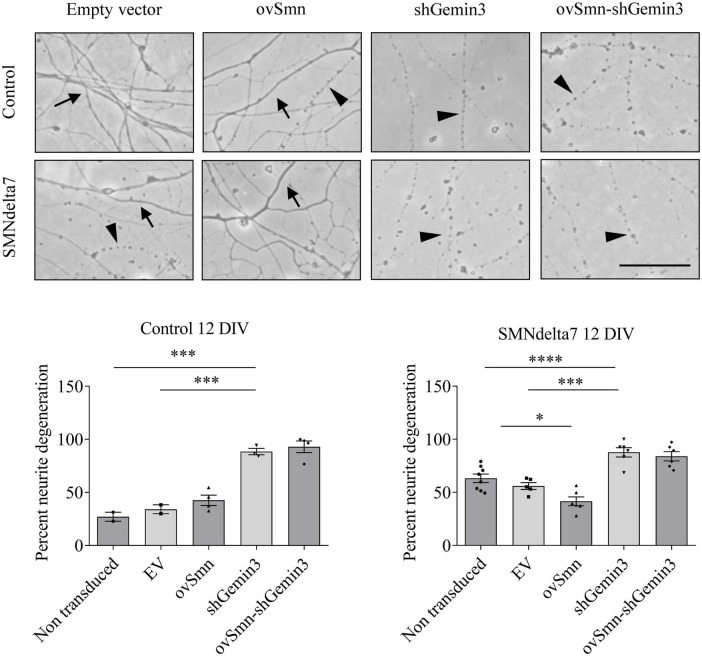
Increased neurite degeneration by Gemin3 knockdown in motoneurons (MNs). Representative phase contrast images of 12 days cultured control and SMNdelta7 MNs transduced with lentiviral particles containing ovSMN, shGemin3, ovSMN plus shGemin3, or empty vector (EV) constructs. Scale bar, 15 μm. Arrows indicate non-degenerating neurites and arrowheads signal degenerated neurites (swelling and blebbing). Graph values are the mean of the percentage of degenerating neurites for each condition of nine wells from three independent experiments ± SEM. Asterisks indicate significant differences using one-way Anova with Tukey’s multiple comparisons post-test (*p < 0.05, ^***^p < 0.001, ^****^p < 0.0001).

To further evaluate the neurodegeneration process occurring in SMA MNs, we next explored neurite degeneration in cultured mouse MNs (control and SMNdelta7). The percentage of degenerating neurites (swelling and blebbing) was considered with respect to the total number of neurites in the microscope field. To determine the presence of neurite degeneration in SMNdelta7 MNs, cells were isolated from 12.5 days embryos and transduced with lentivirus carrying shGemin3 or ovSMN or shGemin3 plus ovSMN or EV or left untreated. After 12 days in culture, SMNdelta7 non-transduced cultures showed an increase of neurite degeneration compared to the control non-transduced condition (63.20 ± 3.98 and 27.03 ± 4.22, *p* = 0.0028, respectively), indicating that SMA MNs displayed neurite degeneration *in vitro*. Nevertheless, when SMN protein was increased (SMNdelta7 ovSMN condition) we observed a reduction in the percentage of neurite degeneration (41.56 ± 4.117) compared to non-transduced (*p* = 0.0051) or EV (55.88 ± 3.197; *p* = 0.0261) controls, suggesting that neurite degeneration in SMA cells was the consequence of SMN reduced protein levels. Interestingly, endogenous knockdown of Gemin3 protein caused a significant increase of neurite degeneration in control (88.49 ± 2.99, *p* = 0.0003) and SMNdelta7 (87.69 ± 4.407, *p* = 0.0002), compared to their respective EV controls. Nevertheless, overexpression of SMN did not prevent neurite degeneration caused by Gemin3 reduction (Figure 7).

## Discussion

The analysis of cellular and molecular mechanisms altered by SMN depletion will provide new evidence to identify targets for the development of complementary or new therapies to prevent SMA neurodegeneration. In the present work, we studied the survival pathway NF-κB and the member of the SMN complex Gemin3 in SMA models. Our results indicated that both NF-κB and Gemin3 are compromised in SMN-reduced MNs, suggesting their contribution to the degeneration mechanisms in these cells.

IKKβ is responsible for the activation of the canonical NF-κB pathway, which involves the nuclear translocation of RelA transcription factor (Li et al., 1999). Previous results showed that IKKβ was reduced in cultured MNs isolated from a severe SMA mouse model (Arumugam et al., 2018). According to these observations, our results indicated that MNs, spinal cord, muscle, and fibroblasts from SMA models exhibited IKKβ reduction. Therefore, decreased IKKβ protein level appears to be a common characteristic in SMN-reduced cells. Nevertheless, RelA protein was reduced in SMA MNs, but not in SMA fibroblasts or muscle, showing differences between SMA cell types. These observations may reflect the complex regulation of the NF-κB pathway. In the nervous system, neuronal activation of NF-κB is critical for cell survival and differentiation, whereas non-neuronal cell activation of NF-κB has a detrimental effect on neurons, triggering inflammatory and cell death mechanisms (Mincheva-Tasheva and Soler, 2013). For instance, increased phosphorylation and upregulation of NF-κB was described in SMN-depleted macrophages (Ando et al., 2020) and human iPSC-derived SMA astrocytes (Allison et al., 2022), suggesting that SMN protein may regulate the activation of these cells and the inflammatory response in SMA. In cultured MNs, pharmacological inhibition of NF-κB pathway or endogenous RelA knockdown reduces SMN level (Arumugam et al., 2018), suggesting an active role of NF-κB in SMN expression in these cells. These differences between cell types suggest the need to consider all of the types when designing therapeutics for SMA that involve modulators of inflammatory or survival intracellular pathways.

Mechanistic studies showed that the member of the SMN complex Gemin3 is a critical co-factor of TAK1 (TGF-β-activated kinase 1)-mediated activation of IKKβ (Shin et al., 2014). Gemin3 interaction with TAK1 enhanced IKKβ phosphorylation and Gemin3 increase produced an overactivation of TAK1, generating a positive feedback loop that activates the NF-κB pathway (Shin et al., 2014, 2015). Our results indicated that Gemin3 protein level is reduced in SMA MNs, but not in SMA fibroblasts or muscle tissue. Moreover, we observed that endogenous reduction of Gemin3 decreased IKKβ and RelA proteins in cultured MNs, indicating NF-κB pathway modulation by Gemin3 in these cells. Immunofluorescence experiments point out a notable decline of Gemin3 in SMA MN neurites. In neurons, granules containing SMN and Gemin3 are distributed throughout the axons to the growth cone and are associated to Gemin2 and heterogeneous nuclear ribonucleoprotein R (hnRNPR) (Zhang et al., 2006). These axonal complexes diverge from those existing in the cell soma. Their function in the axonal compartment may be neuron-specific, different from that in the cell body, and can be related to the modulation of local protein translation at the distal end of MNs (Zhang et al., 2006; Talbot and Davies, 2008; Ottesen et al., 2018).

Survival motor neuron knockdown in HeLa cells reduced protein level of some members of the SMN complex, including Gemin2 and Gemin3, but Gemin3 knockdown did not modify SMN level in these cells (Shpargel and Matera, 2005). Accordingly, in SMN-reduced MNs we observed a decreased Gemin3 level. Nevertheless, our results showed that Gemin3 knockdown reduced SMN protein in control and SMA MNs. In addition, over-expression of SMN increased Gemin3 protein. These results indicated that SMN and Gemin3 levels are interdependent in MNs. SMN and Gemin3 interdependence may be specific to particular tissues and/or neural circuits. In SMN-deficient *C*. *elegans* model pharyngeal pumping rate defects were not ameliorated after *mel-46* (*C. elegans* Gemin3 ortholog) increase, nevertheless, neuromuscular signaling was improved by partially restoring levels and localization of synaptic proteins (O’Hern et al., 2017).

Alterations of Gemin3 have been associated to other diseases. Gemin3 has a functional relationship with Amyotrophic Lateral Sclerosis (ALS)-linked proteins, reinforcing the link between ALS and SMA pathologies. Disruption of either TDP-43 or FUS enhances defects associated with Gemin3 loss-of-function, and Sod1 depletion is responsible of motor decline in Gemin3 mutant *Drosophila* at a late stage in adult life (Cacciottolo et al., 2019). Both SMN and Gemin3 levels are often reduced in SMA patients (Helmken et al., 2003). An inverse correlation between the amount of SMN interacting proteins (such as Gemin3) and the clinical severity of the disease has been observed in SMA lymphoblastoid cell lines (Helmken et al., 2003). Several studies demonstrated a critical role of Gemin3 in motor system and development. In Drosophila, endogenous reduction of Gemin3 in neurons or muscles produced loss of mobility and a flightless phenotype (Shpargel et al., 2009; Curmi and Cauchi, 2018). Additionally, Gemin3 mutations are larval lethal and co-deplete SMN; Gemin3 overexpression rescues lethality but overexpression of SMN does not, suggesting that loss of SMN is not the primary cause of death in the Drosophila model (Shpargel et al., 2009). Moreover, the analysis of the rescuing effect of MEL-46 (Gemin3 ortholog) restoration in a SMN-deficient *C. elegans* model showed that it was not associated to an increase of SMN (O’Hern et al., 2017). In our study of Gemin3 knockdown in cultured MNs, Gemin3 silencing provoked SMN reduction and increased percentage of neurite degeneration. SMN overexpression did not prevent neurite degeneration in Gemin3 reduced cells, reinforcing the role of Gemin3 in MNs homeostasis.

Subcellular transcriptome analysis of SMN-reduced MNs showed no modifications of *RelA*, *Gemin3*, and *IKK*β mRNA in the somatodendritic compartment (Saal et al., 2014). Our results indicated that *RelA* and *Gemin3* mRNA level was increased or not modified in SMA differentiated MNs. These observations suggest that RelA and Gemin3 protein reduction in SMA MNs may be related to protein degradation and/or alterations of *RelA* and *Gemin3* transcripts translation. For instance, some neuropathological conditions produce Gemin3 proteolysis. Infection of cultured cells with poliovirus results in the specific cleavage of Gemin3 by a virus-encoded proteinase (Almstead and Sarnow, 2007) and activation of the calcium-dependent protease calpain, mediates Gemin3 and SMN proteolysis (Walker et al., 2008; de la Fuente et al., 2018). Additionally, SMN is a ribosome-associated protein that functions as a regulator of translation of mRNAs characterized by specific sequence features and linked to the pathogenesis of SMA (Lauria et al., 2020). Moreover, a direct RNA-SMN interaction as a novel mechanism to initiate the cascade of events leading to the execution of SMN specific functions has been proposed. SMN preferentially selects RNA targets through interactions with sequence and structural motifs. Therefore, in addition to its canonical role in ribonucleoprotein biogenesis, these molecular mechanisms suggest a direct implication of SMN in the control of translation of specific SMA-related transcripts (Ottesen et al., 2018).

In summary, the present study describes decreased levels of two members of the NF-κB survival pathway, IKKβ and RelA, in SMA isolated MNs *in vitro* and mouse spinal cord (neural tissue including neurons and non-neuronal cells). However, these changes were not apparent in SMA fibroblasts or muscle, the non-neural tissues analyzed. Gemin3, a member of the SMN complex, was also reduced in SMA isolated MNs and spinal cord. Within this framework, an important aspect to consider is that Gemin3 reduction causes neurite degeneration that cannot be rescued by SMN increase. In addition, Gemin3 reduction in SMA cells may contribute to degeneration through NF-κB inhibition, exacerbating SMA phenotype. Gemin3 may act as a modifying factor for SMA disease and studies of Gemin3 restoration in SMN-reduced MNs will contribute to understand its therapeutic significance.

## Data availability statement

The original contributions presented in this study are included in the article/supplementary material, further inquiries can be directed to the corresponding author.

## Ethics statement

The animal study was reviewed and approved by University of Lleida Advisory Committee on Animal Services (CEEA02-01/17).

## Author contributions

MPM: conceptualization, investigation, formal analysis, writing—original draft, and visualization. AS and MB: investigation, formal Analysis, and validation. AG and RS: conceptualization, supervision, writing—original draft, reviewing, visualization, and funding acquisition. All authors contributed to the article and approved the submitted version.
